# Involvement of Cholesterol Metabolic Pathways in Recovery from Noise-Induced Hearing Loss

**DOI:** 10.1155/2020/6235948

**Published:** 2020-06-12

**Authors:** Na Sai, Xi Shi, Yan Zhang, Qing-qing Jiang, Fei Ji, Shuo-long Yuan, Wei Sun, Wei-Wei Guo, Shi-Ming Yang, Wei-Ju Han

**Affiliations:** ^1^College of Otolaryngology Head and Neck Surgery, Chinese PLA General Hospital, Beijing 100853, China; ^2^National Clinical Research Center for Otolaryngologic Diseases, Beijing, China; ^3^Key Lab of Hearing Science, Ministry of Education, China; ^4^Beijing Key Lab of Hearing Impairment for Prevention and Treatment, Beijing, China; ^5^Clinical Hearing Center of Affiliated Hospital of Xuzhou Medical College, Xuzhou, China; ^6^Department of Otorhinolaryngology Head and Neck Surgery, The First Hospital of Jilin University, Changchun, Jilin 130021, China; ^7^Department of Communicative Disorders and Sciences, Center for Hearing and Deafness, The State University of New York at Buffalo, Buffalo, New York, USA

## Abstract

The objective of this study was to explore the molecular mechanisms of acute noise-induced hearing loss and recovery of steady-state noise-induced hearing loss using miniature pigs. We used miniature pigs exposed to white noise at 120 dB (A) as a model. Auditory brainstem response (ABR) measurements were made before noise exposure, 1 day and 7 days after noise exposure. Proteomic Isobaric Tags for Relative and Absolute Quantification (iTRAQ) was used to observe changes in proteins of the miniature pig inner ear following noise exposure. Western blot and immunofluorescence were performed for further quantitative and qualitative analysis of proteomic changes. The average ABR-click threshold of miniature pigs before noise exposure, 1 day and 7 days after noise exposure, were 39.4 dB SPL, 67.1 dB SPL, and 50.8 dB SPL, respectively. In total, 2,158 proteins were identified using iTRAQ. Both gene ontology and Kyoto Encyclopedia of Genes and Genomes (KEGG) database analyses showed that immune and metabolic pathways were prominently involved during the impairment stage of acute hearing loss. During the recovery stage of acute hearing loss, most differentially expressed proteins were related to cholesterol metabolism. Western blot and immunofluorescence showed accumulation of reactive oxygen species and nuclear translocation of NF-*κ*B (p65) in the hair cells of miniature pig inner ears during the acute hearing loss stage after noise exposure. Nuclear translocation of NF-*κ*B (p65) may be associated with overexpression of downstream inflammatory factors. Apolipoprotein (Apo) A1 and Apo E were significantly upregulated during the recovery stage of hearing loss and may be related to activation of cholesterol metabolic pathways. This is the first study to use proteomics analysis to analyze the molecular mechanisms of acute noise-induced hearing loss and its recovery in a large animal model (miniature pigs). Our results showed that activation of metabolic, inflammatory, and innate immunity pathways may be involved in acute noise-induced hearing loss, while cholesterol metabolic pathways may play an important role in recovery of hearing ability following noise-induced hearing loss.

## 1. Introduction

Noise-induced hearing loss (NIHL) is the most common form of nonhereditary sensorineural hearing loss, the incidence which is increasing annually. An epidemiological survey from 2005 showed that, worldwide, roughly 16% of cases of adult hearing loss were caused primarily by noise overexposure at work [1]. A recent investigation of American adults under 70 years old was performed by the Centers for Disease Control. The results showed that there are roughly 26 million people with NIHL, with prevalence rate of 15%. Furthermore, over 16% of American teenagers (12–19 years old) were shown to have hearing loss caused by excessive noise exposure [2]. NIHL has emerged as a heavy burden to daily communication for patients and for their physical and mental health.

Hair cells in the cochlea play a critical role in converting mechanical sound waves into neural signals for hearing [3–5]. Previous studies using mouse models showed that acoustic injury of the auditory system is caused by multiple factors, and most of the hearing loss induced by noise, different ototoxic drugs, infection, or aging are caused by the hair cells damage [6–13]. Impulse noise (>140 dB SPL) causes hearing loss primarily through mechanical damage of hair cells [14], while steady noise causes metabolic damage of hair cells [15]. However, the complex molecular pathways involved in NIHL remain incompletely understood.

Miniature pigs, which share numerous similarities with humans in terms of inner ear morphology and hearing function, represent a novel large animal model for studying NIHL. Except for primates, pigs and humans have the closest evolutionary relationship, sharing similarities in genetic, anatomical, and physiological factors. The structure of the pig inner ear is very similar to that of humans, and the size of the pig cochlear scala tympani is mostly the same as in humans [16, 17]. These observations suggest that the nerve nuclei of the entire auditory pathway of pigs are highly similar to those of humans. Therefore, the pig model is more suitable than rodents for studying NIHL.

Proteomic Isobaric Tags for Relative and Absolute Quantification (iTRAQ) has been used to identify abnormal protein expression in different diseases [18–20]. In the present study, we aimed to perform proteomic analysis of miniature pig cochleae as pertaining to acoustic trauma. Continuous stimulation with 120 dB (A) white noise was adopted to establish a stable model of NIHL [21]. iTRAQ was used to assay the comparative proteomics of miniature pig inner ears under conditions of noise exposure vs. no noise exposure. Gene ontology (GO) and Kyoto Encyclopedia of Genes and Genomes (KEGG) database analyses of differentially expressed proteins showed that immune pathways may play a key role in the development of NIHL. Major inflammatory factors enriched in KEGG analysis were validated by Western blot and immunofluorescence in noise-exposed pigs. Our results showed that within 24 h of noise exposure, there was significant upregulation and nuclear translocation of NF-*κ*B (p65) in hair cells. NF-*κ*B is a key transcription factor involved in inflammatory signaling pathways, responsible for initiation of transcription of downstream inflammatory factors.

## 2. Materials and Methods

### 2.1. Animals

Healthy miniature pigs (2–3 months, male and female, ~5 kg) were from Zhuozhou Kangning Miniature Pig Cultivation Company (Zhuozhou, China). All animals underwent baseline hearing evaluation. Procedures involving the use and care of animals were supported and controlled by the local ethics committee in compliance with institutional animal protection regulations.

### 2.2. Experimental Procedures

After baseline hearing evaluation, pigs were randomly assigned to a noise exposure group or the control group (no noise exposure). Animals in the noise groups were subjected to noise exposure and scheduled hearing evaluations. Their cochleae were collected for proteomics analyses and pathological analysis at defined time points. Animals in the control group were subjected to the same protocol as in the noise groups except for noise exposure.

Data were objectively measured and analyzed independently by two individual researchers. All animals in the noise exposure groups were compared with corresponding controls, and pigs were randomly assigned to either the noise groups or control group. There were no animal deaths because of attrition, and no data were excluded from analysis.

### 2.3. Noise Exposure

Animals in the noise groups were placed in a wire mesh cage and exposed to white noise at 120 dB (A) for 3 h on 2 consecutive days. The white noise signal was routed through an attenuator (PA5 TDT, Alachua, FL, USA) and a power amplifier (MF-1201 MOSTET, ATech) to a loudspeaker (Aijie Audio Equipment Factory) which was positioned at 20 cm above the animal's head. The noise level at the position of the animal's head in the sound field was calibrated using a sound level meter (Brüel & Kjær, 2250L, Denmark), a preamplifier (RA4PA, 4-channel, TDT), and a condenser microphone (RA4LI, TDT). This noise exposure regime can cause permanent loss in cochlear sensitivity.

### 2.4. Auditory Brainstem Responses

**Figure 1:**
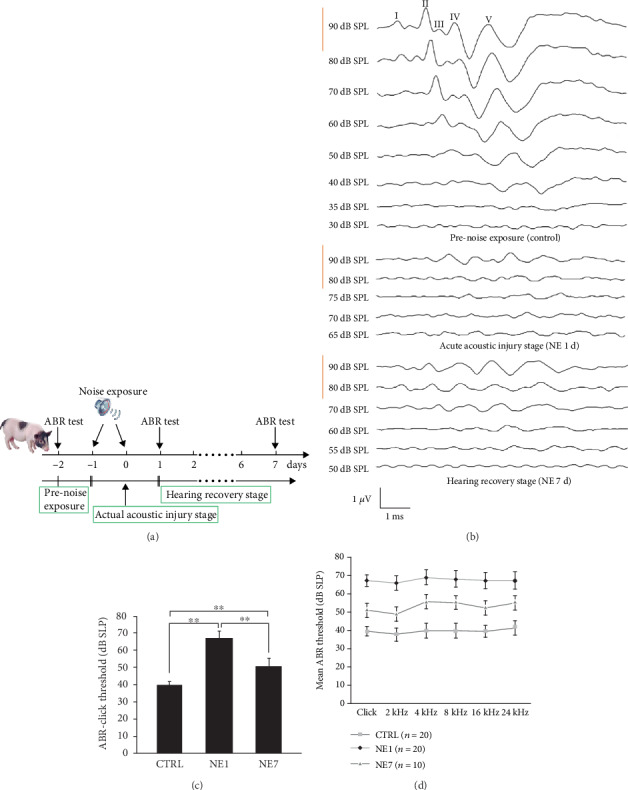
ABR results from three groups of animals. (a) Pattern of hearing changes during different stages of acoustic injury. (b) ABR-click waveforms for the three groups of animals: Top: Normal control individual (CTRL) ABR waveforms, I-V waves are well-differentiated, II- and V-waves are most stable, and this individual ABR threshold is 35 dB SPL. Middle: 1 day after of noise exposure (NE1) testing ABR, the amplitude at 90 dB SPL decreased, and the potential of I-V waves was prolonged. This individual ABR threshold was 70 dB SPL. Bottom: ARB waveforms were tested at 7 days after noise exposure (NE7), and the individual ABR threshold was 55 dB SPL. (c) There was a statistically significant difference in ABR-click thresholds between the three groups of animals. ∗∗ indicates *p* < 0.01, a significant difference (one-way ANOVA, Tukey test). (d) Comparison of ABR-click and tone-burst thresholds in the three groups of animals. *n*: the number of cochleae in each group.

Auditory brainstem response (ABR) measurements were conducted prenoise exposure, 1 day and 7 days postnoise exposure to assess hearing sensitivity of the animals (Figure 1(a)). Each animal was anesthetized with intramuscular injection of Sumianxin (0.1 ml/kg) and 3% pentobarbital sodium (1 ml/kg). Body temperature was maintained at 38°C with a warming blanket. Stainless steel needle electrodes were placed subdermally at the vertex (noninverting input) and behind the stimulated and nonstimulated ears (inverting input and ground, respectively). Each ear was stimulated separately with an open-field sound delivery system positioned at 1 cm from the animal's tested ear. ABRs were induced with clicks and tone bursts at 2, 4, 8, 16, and 24 kHz, generated digitally (SigGen, TDT) using a multifunction processor (RX6, TDT). This was then fed to a programmable attenuator (PA5, TDT), an amplifier (SA1, TDT), and an open-field loudspeaker (MF1-1250, TDT) at 90 dB SPL. The stimulus level was decreased by 10 dB steps until no response was identifiable. The signal was bandpass filtered (100–3000 Hz), amplified (×50,000), and averaged using Tucker Davis Technologies (TDT) System III hardware and SigGen/BioSig version 4.4.1 (TDT, RX6, Alachua, FL, USA) software. Responses were stored and displayed on a computer. The ABR threshold was defined as the lowest stimulus intensity that reliably induced a detectable response.

### 2.5. Cochlear Tissue Collection

Animals were decapitated under deep anesthesia with Sumianxin (0.1 ml/kg) and 3% pentobarbital sodium (1 ml/kg). Cochleae were quickly removed from the skull as previously described [22]. For proteomic and Western blot analyses, cochleae were rinsed with 0.01 M phosphate-buffered saline (PBS), frozen immediately in liquid nitrogen for 10 min, and stored at −80°C. For immunohistological and pathological examinations, cochleae were fixed with 4% paraformaldehyde at 4°C overnight. Cochleae were then dissected in PBS, and the organ of Corti and stria vascularis were collected.

### 2.6. Tissue Protein Extraction and Digestion

Briefly, tissues were harvested and resuspended in 400 *μ*l lysis buffer (8 M urea 50 mM NH_4_HCO_3_, protease inhibitors) and sonicated on ice to extract total protein. The resulting extracts were centrifuged at 10,000 rpm for 30 min at 4°C. Supernatants were collected, and protein concentration was measured using a Bradford assay kit according to the manufacturer's instructions. Protein digestion was performed with the following steps. 200 *μ*g proteins were transferred into ultracentrifugation tube and reduced by adding a final concentration of 10.0 mM dithiothreitol for 60 min at 37°C and then were immediately alkylated by incubating with a final concentration of 20 mM iodoacetamide for 60 min at room temperature in the dark. 100 *μ*L 8 M urea, 50 mM NH4HCO3 were added into tube to clean the proteins in twice. 100 *μ*L 0.5 M triethylammonium bicarbonate (TEAB) were added into tube to exchange the buffer in triple times. Finally, the proteins were digested into the peptides using a trypsin-to-protein ratio of 1 : 50 overnight. The resulting peptides were collected by centrifugation and stored at -80°C.

### 2.7. iTRAQ Labeling and Peptides Prefractionation by High pH Reverse Phase Chromatography

Peptides (100 *μ*g) in 100 mM TEAB from each group were labeled using an 8plex iTRAQ reagents multiplex kit (ABI, Foster City, CA, USA), of which isobaric tags 113 and 114 were for the control group; 115, 116, and 117 were for 1 day postnoise exposure; and 118, 119, and 121 were for 7 days postnoise exposure. In brief, the 8plex iTRAQ reagents were first centrifuged at room temperature and reconstituted with 50 *μ*l isopropyl alcohol to dissolve the iTRAQ labeling reagent. iTRAQ labeling reagents were added to the corresponding peptide samples and were allowed to react at room temperature for 1 h. A total of 100 *μ*l of water was added to prevent the labeling reaction. One aliquot of each sample was analyzed by MS for the test of labeling efficiency. A total of eight sample groups were pooled and vacuum-dried. Each pool of mixed peptides was lyophilized and dissolved in solution A (2% acetonitrile, pH 10, pH adjusted with ammonium hydroxide). Samples were then loaded onto a reverse-phase column (C18 5 *μ*m 4.6 × 250 mm, waters) and eluted using a step linear elution program: 5%–35% buffer B at flow rate of 0.7 ml/min (98% acetonitrile, without pH adjustment, solution B) for 30 min, 35%–95% buffer B for 2 min, 95% buffer B for 5 min, and 95%–5% buffer B for 2 min. Samples were collected every 1.5 min. The collected fractions (about 40) were finally combined into 10 pools and desalted on C18 Cartridges (Empore™ standard density SPE C18 Cartridges, bed I.D. 7 mm, 3 ml volume; Sigma, St. Louis, MO, USA).

### 2.8. LC-Electrospray Ionization-MS/MS Analysis

We referred to the method of Wang et al. [23]. NanoLC-MS/MS experiments were performed with a Q-Exactive HF mass spectrometer (Thermo Fisher Scientific, Waltham, MA, USA) coupled with a nano-high-performance liquid chromatography (UltiMate 3000 LC Dionex; Thermo Fisher Scientific) system. iTRAQ-labeling peptides were loaded onto a C18-reversed phase column (3 *μ*m C18 resin, 0.1 × 20 mm) and separated on an analytical column (1.9 *μ*m C18 resin, 0.15 × 120 mm; Dr. Maisch GmbH, Ammerbuch, Germany) using mobile phase A: 0.5% formic acid (FA)/H_2_O and B: 0.5% FA/ACN at a flow rate of 600 nl/min, using a 90 min gradient. Spectra were acquired in data-dependent mode. The 20 most intensive ions were selected for MS scanning (300–1400 *m*/*z*, 120,000 resolution at *m*/*z* 400, accumulation of 3.0*E*6 ions for a maximum of 80 ms, one microscan). The isolation window was 1.6 m/z, and MS/MS spectra were measured at resolution of 15,000 at *m*/*z* 400. Dynamic precursor exclusion was allowed for 60 s after each MS/MS spectrum measurement. Normalized collision energy was 30%.

### 2.9. MS Data Analysis

Raw MS data were processed using Proteome Discoverer 1.4 (ver. 1.4.0.288; Thermo Fisher Scientific). Briefly, peptide identification was performed with Sequest HT search engine against a Uniprot Human Complete Proteome database supplemented with all frequently observed MS contaminants. The following options were used to identify the proteins: Peptide mass tolerance = ±15 ppm, MS/MS tolerance = 0.2 Da, enzyme = trypsin, missed cleavage = 2; fixed modification: iTRAQ 8-plex (K) and iTRAQ 8-plex (N-term), variable modification: oxidation (M), database pattern = decoy. The peptide confidence was set to a high level (*q*-value <0.01) for peptide filtering. Quantification experimental bias was set as normalize on total peptide amount. Up- or downregulated proteins with 1.2-fold changes were selected as being differentially expressed.

### 2.10. Bioinformatics Analysis

Gene ontology (GO) enrichment analysis (http://www.geneontology.org) of differentially expressed proteins with 1.2-fold changes was performed to classify molecular functions, cellular components, and biological processes. Interactions among these proteins pertaining to biological pathways were determined using Pathway Studio software and the ResNet database (KEGG) to better understand them in relation to the published literature. The Pathway Maps tool was used to enrich the pathways, and *P* values were calculated based on a hypergeometric distribution, with the default database used as the background. Significant pathway enrichment was defined as corrected FDR of *P* ≤ 0.05, and proteins with ≥1.2-fold changes were considered differentially abundant proteins.

### 2.11. Immunohistochemistry

Immunohistochemistry was used to examine changes in expression of NF-*κ*B (p65) and Apolipoprotein (Apo) A1 in cochleae. Animals were sacrificed on days 1 or 7 postnoise exposure. Cochleae were fixed with 4% paraformaldehyde at 4°C overnight. After dissection in 0.01 M PBS, the organ of Corti and stria vascularis were collected. Tissues were then permeabilized with 0.25% Triton X-100 in PBS for 30 min, blocked with 5% goat serum in PBS for 30 min, and incubated overnight at 4°C with primary antibody at concentrations recommended by the manufacturer. Tissues were then rinsed with PBS (three times), incubated with secondary antibody at room temperature for 1 h, and counterstained with DAPI for 10 min.

For the noise exposure groups, six cochleae each were used for NF-*κ*B (p65) (6956, Cell Signaling Technology, Inc) and Apo A1 (Abcam, ab64308) staining. Cochleae from six additional animals that were not subjected to noise exposure were used as controls. Several sections of tissue from cochleae were stained only with secondary antibodies to assess nonspecific staining.

Fluorescence was visualized under a confocal microscope (Zeiss LSM780 laser scanning confocal image system) as previously described [24]. The numbers of different stained hair cells were counted for further quantitative analysis as previously described [25].

### 2.12. Detection of Intracellular Reactive Oxygen Species

The increasing fluorescence intensity of 2′,7′-dichlorofluorescein (DCF) was used to measure the generation of intracellular reactive oxygen species (ROS). The reagent 2′,7′-dichlorodihydorofluorescein diacetate (DCFH-DA; Sigma-Aldrich, USA) can enter the cell, where the diacetate group is cleaved off by intracellular esterase. The resulting DCFH is retained in the cytoplasm and oxidized to DCF by ROS. The organ of Corti was dissected from the inner ears of normal control animals and from animals on day 1 after noise exposure and incubated in 200 *μ*l DCFH-DA working solution (20 mM) at 37°C for 30 min. Hair cells were observed under a confocal microscope (Zeiss LSM780 laser scanning confocal image system). The fluorescence of DCF was monitored at excitation and emission wavelengths of 485 nm and 530 nm, respectively.

### 2.13. Western Blot

Tissues from pig cochleae were lysed with RIPA. Western blotting was performed similarly to previous studies [26]. Briefly, proteins separated by SDS-PAGE were transferred to polyvinylidene fluoride membranes (Millipore, Bedford, MA). Membranes were treated with anti-NF-*κ*B p65 (6956, Cell Signaling Technology, Inc) and Apo A1 (Abcam, ab64308) antibodies. Each antibody preparation was diluted in 5% skim milk, and protein bands were visualized using an ECL plus chemiluminescence detection system (WBKLS0500, Millipore, USA) and photographed.

### 2.14. Statistical Analysis

Data are presented as mean ± standard deviation. Average ABR thresholds and immunoreactivity and protein expression obtained before and 1 and 7 days after noise exposure were compared using one-way ANOVA. If significant differences were observed from one-way ANOVA, the Tukey test or Kruskal-Wallis test was performed to delineate the nature of the differences using SPSS version 19.0 (SPSS, Inc., Chicago, IL, USA). *P* ≤ 0.05 was considered statistically significant. The ratio of number of cells with nuclear malformations (fragmented or condensed) to total number of cells was calculated.

## 3. Results

### 3.1. Noise Exposure Causes Loss in Cochlear Sensitivity

We established a swine model of permanent hearing loss induced by noise exposure. One-month-old pigs (5 kg) with normal hearing ability (Figure 1(b) top panel) were exposed to 120 dB (A) white noise for 3 h on 2 consecutive days. ABR measurements to monitor acute hearing loss and hearing recovery were performed on days 1 and 7 after noise exposure (Figure 1(a)). Noise exposure caused loss in cochlear sensitivity. All experimental animals underwent ABR-click to test baseline hearing level before noise treatment. ABR-click and tone burst were performed in three groups of animals (prenoise exposure and days 1 and 7 after noise exposure). The different ABR-click waveforms in the three groups are shown in Figure 1(b). There were 10 pigs (*n* = 20 ears) in the control group, 10 pigs (*n* = 20 ears) in the 1 day postnoise exposure group, and 5 pigs (*n* = 10 ears) in the 7 days postnoise exposure group. The average ABR-click threshold was 39.4 ± 2.6 dB SPL in prenoise exposure animals, 67.1 ± 4.1 dB SPL in 1 day postnoise exposure animals, and 50.8 ± 4.7 dB SPL in the 7 days postnoise exposure group (Figure 1(c)). Hearing loss was most severe at 4 kHz, and hearing loss at high frequency was more severe than at low frequency, which was consistent with human hearing performance in acute NIHL [15]. Average hearing threshold could be recovered to 14 ± 4.9 dB SPL higher than the normal level after 7 days from noise exposure, and hearing loss recovery from 4 kHz and higher frequencies was worse compared with the low frequency (Figure 1(d)).

### 3.2. Comparative Proteomic Analysis of Cochleae Prenoise Exposure and during the Acute and Recovery Stages Postnoise Exposure

**Figure 2:**
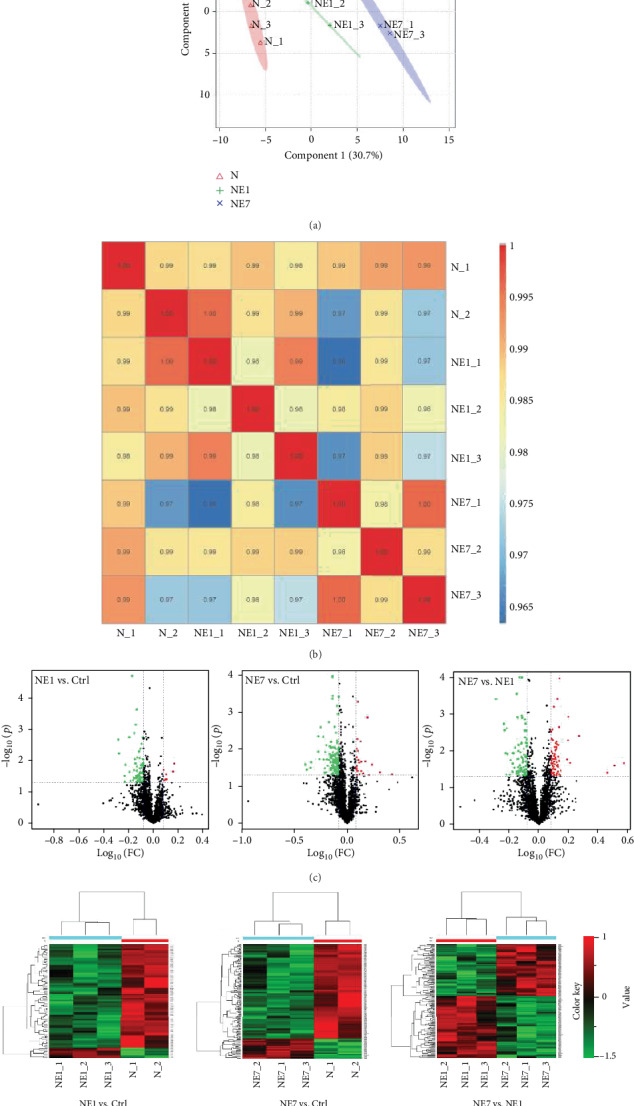
Proteomic clustering reveals three distinct groups. (a) Principal component analysis (PCA) of the three groups: normal control group (N), 1 day postnoise exposure group (NE1), and 7 days postnoise exposure group (NE7). (b) Correlation analysis between control and noise exposure pig cochleae. (c) Volcano analysis showing upregulated (red) and downregulated (green) proteins from NE1 vs. Ctrl (left), NE7 vs. Ctrl (middle), and NE1 vs. N7 (right). (d) Heat map analysis showing upregulated (red) and downregulated (green) proteins from NE1 vs. Ctrl (left), NE7 vs. Ctrl (middle), and NE1 vs. NE7 (right).

Proteomic data were collected from miniature pigs of the control (*n* = 2), 1 day postnoise exposure (*n* = 3), and 7 days postnoise exposure groups (*n* = 3). Principal component analysis showed good distribution between the three groups (Ctrl, NE1, and NE7) (Figure 2(a)). Correlation analyses of samples from the same groups were over 98% (Figure 2(b)), indicating samples from the same groups had high similarity. Changes in protein expression induced by noise exposure are shown in a volcano plot and heat map analysis in Figures 2(c) and 2(d). 68 proteins were downregulated (green) and 7 proteins were upregulated (red) between the 1 day postnoise exposure and control groups. Between the 7 days postnoise exposure and control groups, 125 proteins were downregulated (green) and 26 proteins were upregulated (red). Between the 1 day postnoise exposure and 7 days postnoise exposure groups, 73 proteins were upregulated (red) and 88 proteins were downregulated (green).

### 3.3. Immune and Oxidative Stress Are Triggered during the Acute Acoustic Injury Period

**Figure 3:**
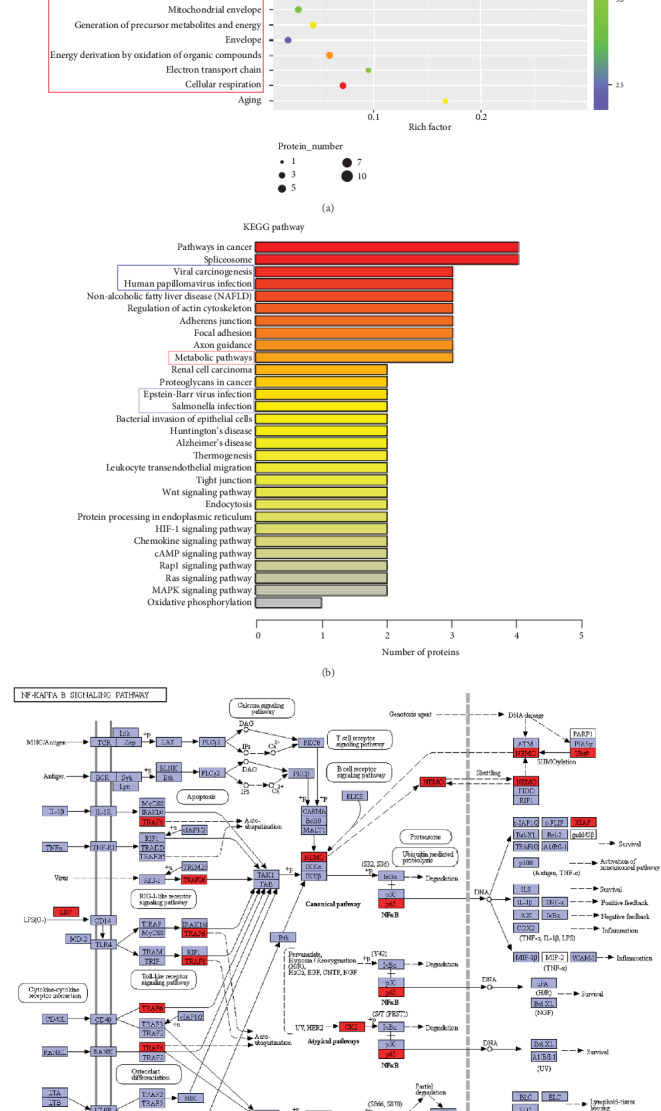
Activation of metabolic (red frame) and immune (blue frame) pathways in pig cochleae were prominent during the acute response to noise exposure. GO (a) and KEGG (b) analyses show that metabolic (red frame) and immune (blue frame) function were prominently enriched among proteins that changed in levels between control (Ctrl) and day 1 after noise exposure (NE1) pig inner ears. (c) The NF-*κ*B (p65) signaling pathway was primarily activated at day 1 after noise exposure (NE1) in pig inner ears.

To identify physiological changes in pig cochleae during the acute period following noise exposure, we compared dysregulated proteins between the NE1 and Ctrl groups. GO analysis (Figure 3(a)) showed that oxidative stress (red frame) and immune response (blue frame) were the two groups primarily annotated in response to noise-induced cochlear damage. Consistent observations were made following analysis of enriched proteins in KEGG analysis (Figures 3(b) and 3(c)). Moreover, we found that p65, part of the NF-*κ*B transcription factor family, was increased following noise exposure and contributed to inducing the acute period of cochlear damage (Figure 3(c)).

### 3.4. Accumulation of ROS and Activation of NF-*κ*B Signaling in Pig Hair Cells during the Acute Acoustic Injury Period

**Figure 4:**
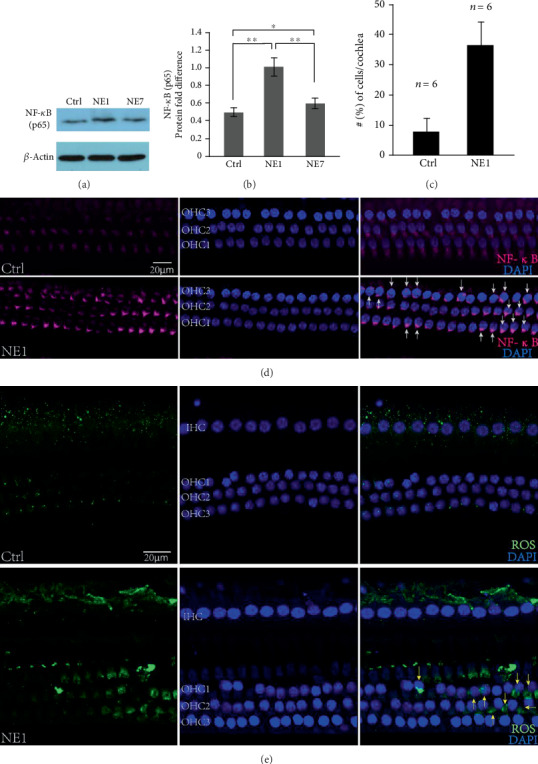
Accumulation of ROS and activation of NF-*κ*B signaling were validated in NE1 pig inner ears. Upregulated NF-*κ*B (p65) protein level were detected by Western blot (a) and analyzed statistically using Image Gallery software (b) based on (a). Asterisk indicates a significant difference (^∗^*p* < 0.05, ^∗∗^*p* < 0.01, one-way ANOVA, Tukey test). (c) Average percentage of outer hair cells per cochlea that exhibited NF-*κ*B (p65) translocation into the nucleus. (d) Immunofluorescence image showing nuclear translocation of NF-*κ*B (p65) in outer hair cells (white arrow) 1 day after noise exposure. (e) Immunofluorescence image showing accumulation of ROS in hair cells (yellow arrow) 1 day after noise exposure. *n*: number of cochleae.

**Figure 5:**
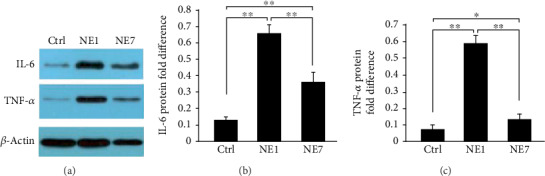
IL-6 and TNF-*α* were upregulated in the NE1 compared with the control group, and downregulated in the NE7, as determined by Western blot (a). Statistical analysis using Image Gallery software (b, c) based on (a). Asterisk indicates a significant difference (^∗^*p* < 0.05, ^∗∗^*p* < 0.01, one-way ANOVA, Tukey test).

Protein expression of NF-*κ*B (p65) was examined by Western blot. Noise exposure significantly increased p65 on 1 day after noise exposure, which gradually recovered by 7 days after noise exposure (Figures 4(a)–4(c)). The subcellular location of p65 in cochleae was examined using immunofluorescence. Nuclear translocation of NF-*κ*B (p65) was detected in outer hair cells after noise exposure (Figure 4(d)). Moreover, IL-6 and TNF-*α* were detected by Western blot and showing they are upregulated in NE-1 and partly decreased in NE-7 (Figures 5(a)–5(c)). The initiation of downstream inflammatory factors may be because of a significant accumulation of ROS in the hair cells on the 1st day after the noise treated (Figure 4(e)).

### 3.5. Cholesterol Metabolism Pathways Are Involved in Recovery from NIHL

**Figure 6:**
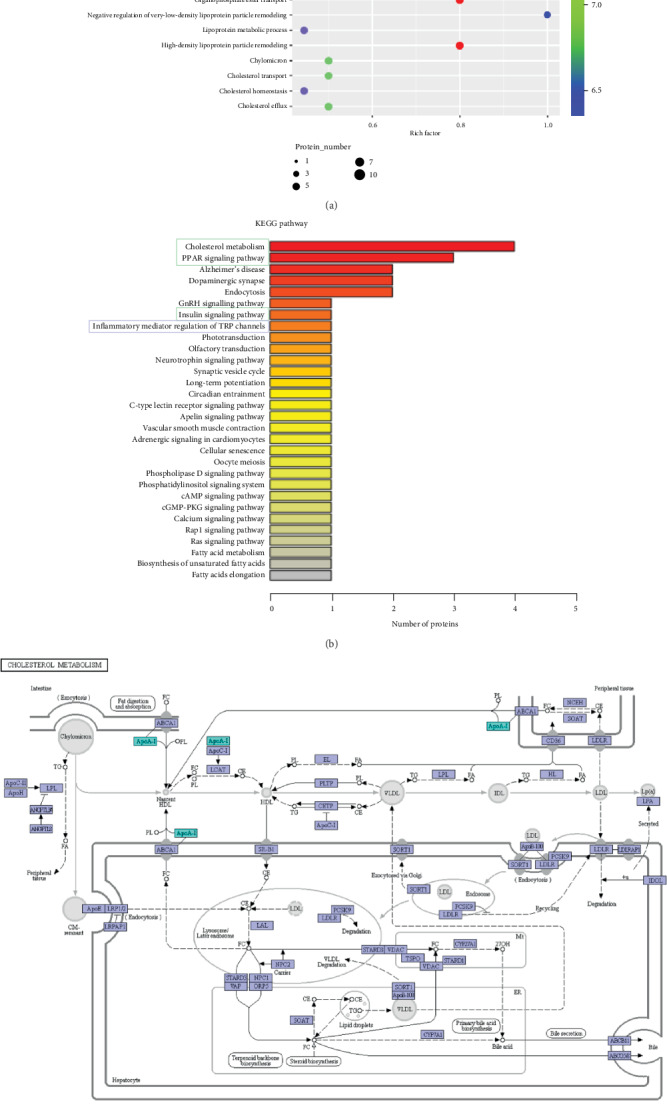
Activation of cholesterol transport pathways in pig cochleae was prominent during the recovery stage after noise exposure. GO (a) and KEGG (b) analyses show cholesterol transport (green frame) function was prominently enriched among proteins that changed in levels between control (Ctrl) and day 7 after noise exposure (NE7) pig inner ears. (c) Cholesterol metabolism signaling pathways were primarily activated in day 7 after noise exposure (NE7) pig inner ears.

**Figure 7:**
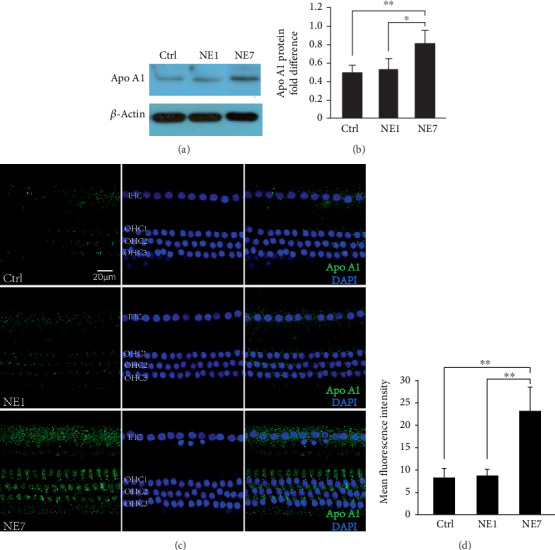
Apo A1 was upregulated in the NE7 group compared with the normal control and NE1 groups, as determined by Western blot (a). Statistical analysis using Image Gallery software (b) based on (a). Asterisk indicates a significant difference (^∗∗^*p* < 0.01, ^∗^*p* < 0.05, one-way ANOVA, Tukey test). (c) Cholesterol metabolic pathways may play an important role in recovery from hearing loss after noise exposure. Immunofluorescence image shows that fluorescence intensity of Apo A1 in hair cells increased at 7 days after noise exposure. Statistical analysis of fluorescence intensity using Image J software (d) based on (c). Asterisk indicates a significant difference (^∗∗^*p* < 0.01, one-way ANOVA, Tukey test).

**Figure 8:**
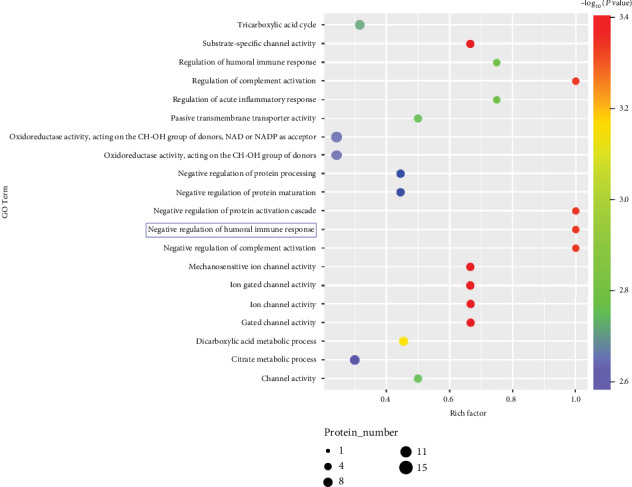
GO analysis shows that immune (blue frame) function were prominently enriched among proteins that changed in levels between day 7 after noise exposure (NE7) and day 1 after noise exposure (NE1) pig cochlea.

To evaluate protein expression during the recovery stage of NIHL, we analyzed dysregulated proteins between the NE7 and Ctrl groups. Both GO (Figure 6(a)) and KEGG (Figure 6(b)) analyses showed that the PPAR and insulin pathways (green frames), involved in cholesterol metabolism, were involved during this stage. We also found that Apo AI was increased in NE7 cochleae (Figures 6(c) and 7) and may contribute to hearing recovery. Additionally, other proteins involved in inflammation were increased (blue frames). Through comparing NE7 with NE1 group, we found that some of these differential proteins were enriched in the negative regulation of humoral immune response (blue frame) (Figure 8), which suggested negative immune regulation pathways were involved in the hearing recovery stage. These results demonstrate that gene expression differed between stages after noise exposure. During the acute stage, inflammatory and oxidative stress-related pathways were involved in mediating cochlear damage, while during the recovery stage, inner ear damage induced by inflammation was resolved gradually via cholesterol metabolic pathways.

**Figure 9:**
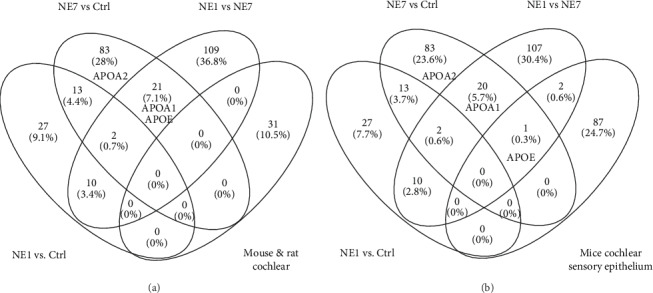
Venn map showing common differentially expressed genes (DEG) between previous and the present study. (a) No common genes were found in pig, mouse, and rat cochleae in different noise-induced hearing loss models. (b) A hearing protective factor, Apo-E, was increased in mouse and rat cochleae.

To determine whether our results were similar to those from prior studies in rodent models, we analyzed these studies and appropriate databases and found that the Apo A and Apo E genes were previously shown to be expressed in adult mice by inner and outer hair cells (Table 1). However, no genes were commonly increased between mouse and rat transcriptomics (Figure 9(a)). Apo E was unique in that it was upregulated in both mouse cochlear sensory epithelium and pig cochleae (Figure 9(b)). These observations demonstrate differences in gene regulation between pigs and rodents, although the cholesterol metabolic pathway may represent a common pathway involved during the hearing recovery stage of NIHL in different animal models.

## 4. Discussion

In the present study, proteomic analysis showed that activation of metabolic and innate immune signaling pathways may be involved in noise-related acute hearing loss. We also detected accumulation of ROS and nuclear translocation of NF-*κ*B (p65) by immunofluorescence which was consistent with previous studies demonstrating that the inner ear produces a large number of free radicals, including ROS and reactive nitrogen species following potent noise stimulation [27]. Endogenous antioxidant enzymes were insufficient to remove excessive free radicals, which resulted in lipid peroxidation and damage to the structure of DNA, proteins, and mitochondria [28, 29]. Many recent studies also showed that the inner ear has strong immune ability. Cumulative evidence has indicated that NF-*κ*B is a key transcription factor driving inflammation and that TNF-*α*, ROS, and NF-*κ*B are inextricably tied together in inflammation and immunity of noise-induced hearing loss [30]. ROS interacts with NF-*κ*B signaling pathways in many ways. However, it is not yet understood how accumulation of ROS activates NF-*κ*B in the cochlea, but it is assumed that ROS influence the activation of NF-*κ*B pathway mainly by inhibiting the phosphorylation of I*κ*B*α* [30, 31]. Moreover, some of NF-*κ*B downstream inflammatory factors, including IL-6 and TNF-*α*, were also detected by Western blot and showed that they are upregulated in NE1 and partly decreased in NE7 (Figures 5(a)–5(c)), which is consistent with the changes of the NF-*κ*B (p65) (Figures 4(a) and 4(b)). A report analyzing cochlear sensory epithelium using RNA sequencing showed that most upregulated genes were related to immunity, inflammation, and defense response after noise exposure [32], indicating that the immune/inflammatory response is an important mechanism in NIHL and the primary reaction of the cochlea to noise stimulation.

We also found that proteins involved in cholesterol metabolism, Apo E and Apo AI, were increased 7 days after noise exposure. Epidemiological evidence indicated that high levels of apoA-1/apoA-2 and Apo-E are associated with protection against atherosclerotic disease and negative regulation of cytokine secretion involved in immune responses. However, the mechanisms involved in these beneficial effects are not well established [33]. A recent study showed that Apo E gene variants may have been associated with sudden sensorineural hearing loss in an Iranian population [34]. These studies indicate that cholesterol metabolism may be important in hearing loss recovery after noise exposure. Moreover, as shown in Table 1, the Apo-E, Apo-AI, and Apo-AII genes were expressed in adult mouse cochlear inner and outer hair cells, indicating that these genes have related biological functions in the inner ear. Notably, the involvement of Apo AI and Apo A II in NIHL has seldom been reported.

RNA transcriptomic analysis has been wildly used in the hearing research fields to identify the differentially expressed genes [35–41]. Compared with previous studies that analyzed RNA transcriptomics, differential genes related to NIHL involve inflammation mediated by chemokines, cytokine pathways of the stress response, and immune pathways enriched by KEGG [32, 42]. These observations are consistent with this study. However, compared with the present study, only a small number of common differentially expressed genes were found in previous molecular profile studies of acoustic trauma in rodent cochleae. Apo-E was a common gene included in both subsets in this study (NE7 vs. Ctrl and NE7 vs. NE1) and in mouse normal cochlear sensory epithelium. However, no genes were found to be commonly expressed in all three species (rats, mice, and pigs) (Figure 9(a)) [33]. Possible explanations are as follows: (1) the noise processing conditions used in this study were different from previous studies; (2) pigs are large animals and may differ significantly from rodents in gene expression patterns; and (3) previous studies used RNA-seq transcriptomics, while proteomic analysis (iTRAQ) was used here. Previous studies showed that the consensus between differential gene expression analyzed by transcriptomics and proteomics under the same experimental conditions was less than 30% [43]. Therefore, it is possible that the results of proteomics analysis obtained herein will not overlap with differential genes from transcriptomics screenings of previous related studies of noise exposure in rodents. This indirectly reflected the necessity and advantages of using large animals and proteomics technology to study the mechanisms of auditory diseases. Large animals are more closely related to humans in terms of gene homology and regulation. In addition, proteins are the ultimate effector molecules for mediating biological processes. Therefore, analysis of disease mechanisms using proteomics combined with large animal models may be promising for future studies.

We showed that cholesterol metabolic pathways are involved in recovery from NIHL. In the inner ear of pigs exposed to noise, both Apo E and Apo AI protein were increased at day 7 and were accompanied with simultaneous 30 dB threshold recovery. Through the previous studies, it has been confirmed that Apo A-I and Apo E were down-regulated by NF-*κ*B activation, while Apo A-I and Apo E expression suppressed NF-*κ*B-mediated inflammation [44]. Apo A-I, as well as Apo E, has been shown to regulate lipid metabolism and inflammation [45–47], and Apo A-I and Apo E exert anti-inflammatory properties that protect against atherosclerosis and other inflammatory diseases [45]. In addition, the absence of them increased inflammation and oxidative stress through activation of NF-*κ*B [47, 48]. In our study, significantly increased NF-*κ*B (p65) were observed in day 1 after noise exposure (Figures 4(a) and 4(b), NE1 vs. Ctrl) and dramatically downregulated in day 7 (Figures 4(a) and 4(b), NE1 vs. NE7), although not yet fully restored to normal levels (Figures 4(a) and 4(b), NE7 vs. Ctrl). It indicated that NF-*κ*B was activated in acute stage after noise exposure (day 1) but gradually recovered in day 7 (Figures 4(a)–4(c)). In addition, Apo A-I was increased in NE7 but not in NE1 cochleae. We speculated that although NF-*κ*B (p65) protein expression are still higher in NE7 compared with that in Ctrl, in fact, the NF-*κ*B-mediated inflammation has been gradually suppressed by ApoA-I. This suggests Apo E and Apo A-I are hearing protective factors involved in alleviating oxidative stress and inflammation induced during the acute phase of noise-mediated hearing impairment (day 1 after noise exposure). More notable was that the involvement of Apo AI in NIHL has not been reported previously. However, the specific mechanism still requires further investigation.

In conclusion, this study further verified that inflammation and oxidative stress are the main causes of noise-related deafness. Induction of cholesterol metabolic pathways may represent an important mechanism for alleviating noise-induced inner ear impairment. We propose that antioxidation and anti-inflammatory therapy may be effective treatment options for clinical NIHL. However, the findings in this study represent a small part of the very complex mechanism underlying NIHL, involving multiple molecular networks. Therefore, there remains much to be learned.

## Figures and Tables

**Table 1:** Expression of ApoA and ApoE genes in adult mice assessed by inner (IHC) and outer Hair cells (OHC).

Probeset ID	IHC (mean ± sd)	OHC (mean ± sd) [OHC replicate]	Fold change (OHC/IHC)	False discovery rate (FDR)
ApoE 17487381	54.65 ± 1.55	43.14 ± 11.82 [65.40]	0.79	0.504
ApoA1 17516901	13.98 ± 0.00	16.00 ± 2.11 [12.85]	1.14	0.536
ApoA2 17219242	10.89 ± 0.00	11.15 ± 0.19 [10.89]	1.02	0.501

## References

[1] Nelson D. I., Nelson R. Y., Concha-Barrientos M., Fingerhut M. (2005). The global burden of occupational noise-induced hearing loss. *American Journal of Industrial Medicine*.

[2] Shargorodsky J., Curhan S. G., Curhan G. C., Eavey R. (2010). Change in prevalence of hearing loss in US adolescents. *JAMA*.

[3] Liu Y., Qi J., Chen X. (2019). Critical role of spectrin in hearing development and deafness. *Science Advances*.

[4] Qi J., Liu Y., Chu C. (2019). A cytoskeleton structure revealed by super-resolution fluorescence imaging in inner ear hair cells. *Cell Discovery*.

[5] Qi J., Zhang L., Tan F. (2020). Espin distribution as revealed by super-resolution microscopy of stereocilia. *American Journal of Translational Research*.

[6] He Z. H., Zou S. Y., Li M. (2020). The nuclear transcription factor FoxG1 affects the sensitivity of mimetic aging hair cells to inflammation by regulating autophagy pathways. *Redox Biology*.

[7] Gao S., Cheng C., Wang M. (2020). Blebbistatin inhibits neomycin-induced apoptosis in hair cell-like HEI-OC-1 cells and in cochlear hair cells. *Frontiers in Cellular Neuroscience*.

[8] Zhang Y., Li W., He Z. (2019). Pre-treatment with Fasudil prevents neomycin-induced hair cell damage by reducing the accumulation of reactive oxygen species. *Frontiers in Molecular Neuroscience*.

[9] Liu W., Xu X., Fan Z. (2019). Wnt signaling activates TP53-induced glycolysis and apoptosis regulator and protects against cisplatin-induced spiral ganglion neuron damage in the mouse cochlea. *Antioxidants & Redox Signaling*.

[10] Zhang S., Zhang Y., Dong Y. (2020). Knock-down of Foxg1 in supporting cells increases the transdifferentiation of supporting cells into hair cells in the neonatal mouse cochlea. *Cellular and Molecular Life Sciences*.

[11] Tan F., Chu C., Qi J. (2019). AAV-ie enables safe and efficient gene transfer to inner ear cells. *Nature Communications*.

[12] He Z., Guo L., Shu Y. (2017). Autophagy protects auditory hair cells against neomycin-induced damage. *Autophagy*.

[13] He Z., Fang Q., Li H. (2019). The role of FOXG1 in the postnatal development and survival of mouse cochlear hair cells. *Neuropharmacology*.

[14] Ayna G., Krysko D. V., Kaczmarek A., Petrovski G., Vandenabeele P., Fesus L. (2012). ATP release from dying autophagic cells and their phagocytosis are crucial for inflammasome activation in macrophages. *PLoS One*.

[15] Li X. (2011). *Pathophysiology of the Cochlea*.

[16] Zhong L. L., Zhang Y., Liang X. J. (2018). Inner ear structure of miniature pigs measured by multi-planar reconstruction techniques. *American Journal of Translational Research*.

[17] Yang S. M. (2016). The miniature pig as an animal model in otological research. *Chinese Journal of Otology*.

[18] Marianowski P., Szymusik I., Malejczyk J., Hibner M., Wielgos M. (2013). Proteomic analysis of eutopic and ectopic endometriotic tissues based on isobaric peptide tags for relative and absolute quantification (iTRAQ) method. *Neuro Endocrinology Letters*.

[19] Fang Q., Zhang Y., Chen X. (2020). Three-dimensional graphene enhances neural stem cell proliferation through metabolic regulation. *Frontiers in Bioengineering and Biotechnology*.

[20] Han S., Xu Y., Sun J. (2020). Isolation and analysis of extracellular vesicles in a Morpho butterfly wing-integrated microvortex biochip. *Biosensors & Bioelectronics*.

[21] Wu J., Han W., Chen X. (2017). Matrix metalloproteinase-2 and -9 contribute to functional integrity and noiseinduced damage to the blood-labyrinth-barrier. *Molecular Medicine Reports*.

[22] Chen L., Guo W., Ren L. (2016). A de novo silencer causes elimination of MITF-M expression and profound hearing loss in pigs. *BMC Biology*.

[23] Wang C., Zhao D., Shah S. Z. A., Yang W., Li C., Yang L. (2017). Proteome analysis of potential synaptic vesicle cycle biomarkers in the cerebrospinal fluid of patients with sporadic Creutzfeldt–Jakob disease. *Molecular Neurobiology*.

[24] Shi X., Wu N., Zhang Y., Guo W., Lin C., Yang S. (2017). Adeno-associated virus transformation into the normal miniature pig and the normal guinea pigs cochlea via scala tympani. *Acta Oto-Laryngologica*.

[25] Hu B. H., Cai Q., Hu Z. (2012). Metalloproteinases and their associated genes contribute to the functional integrity and noise-induced damage in the cochlear sensory epithelium. *The Journal of Neuroscience*.

[26] Shi X., Dong Y., Li Y. (2015). Inflammasome activation in mouse inner ear in response to MCMV induced hearing loss. *Journal of Otology*.

[27] Kopke R. D., Weisskopf P. A., Boone J. L. (2000). Reduction of noise-induced hearing loss using L-NAC and salicylate in the chinchilla. *Hearing Research*.

[28] Rybak L. P., Ravi R., Somani S. M. (1995). Mechanism of protection by diethyldithiocarbamate against cisplatin ototoxicity: antioxidant system. *Fundamental and Applied Toxicology*.

[29] Van Campen L. E., Murphy W. J., Franks J. R., Mathias P. I., Toraason M. A. (2002). Oxidative DNA damage is associated with intense noise exposure in the rat. *Hearing Research*.

[30] Fetoni A. R., Paciello F., Rolesi R., Paludetti G., Troiani D. (2019). Targeting dysregulation of redox homeostasis in noise-induced hearing loss: oxidative stress and ROS signaling. *Free Radical Biology and Medicine*.

[31] Zhang J., Wang X., Vikash V. (2016). ROS and ROS-mediated cellular signaling. *Oxid Med Cell Longev*.

[32] Yang S., Cai Q., Vethanayagam R. R., Wang J., Yang W., Hu B. H. (2016). Immune defense is the primary function associated with the differentially expressed genes in the cochlea following acoustic trauma. *Hearing Research*.

[33] Cimmino G., Ibanez B., Vilahur G. (2009). Up-regulation of reverse cholesterol transport key players and rescue from global inflammation by ApoA-IMilano. *Journal of Cellular and Molecular Medicine*.

[34] Hamidi A. K., Yazdani N., Seyedjavadi K. H. (2019). MTHFR AND ApoE genetic variants association with sudden sensorineural hearing loss. *American Journal of Otolaryngology*.

[35] Zhang S., Zhang Y., Yu P. (2017). Characterization of Lgr5+ progenitor cell transcriptomes after neomycin injury in the neonatal mouse cochleae. *Frontiers in Molecular Neuroscience*.

[36] Guo R., Ma X., Liao M. (2019). Development and application of cochlear implant-based electric-acoustic stimulation of spiral ganglion neurons. *ACS Biomaterials Science & Engineering*.

[37] Zhang Y., Guo L., Lu X. (2018). Characterization of Lgr6+ cells as an enriched population of hair cell progenitors compared to Lgr5+ cells for hair cell generation in the neonatal mouse cochlea. *Frontiers in Molecular Neuroscience*.

[38] Cheng C., Guo L., Lu L. (2017). Characterization of the transcriptomes of Lgr5+ hair cell progenitors and Lgr5− supporting cells in the mouse cochlea. *Frontiers in Molecular Neuroscience*.

[39] Cheng C., Wang Y., Guo L. (2019). Age-related transcriptome changes in Sox2+ supporting cells in the mouse cochlea. *Stem Cell Research & Therapy*.

[40] Tang M., Li J., He L. (2019). Transcriptomic profiling of neural stem cell differentiation on graphene substrates. *Colloids and Surfaces. B, Biointerfaces*.

[41] You D., Guo L., Li W. (2018). Characterization of Wnt and notch-responsive Lgr5+ hair cell progenitors in the striolar region of the neonatal mouse utricle. *Frontiers in Molecular Neuroscience*.

[42] Cai Q., Vethanayagam R. R., Yang S. (2014). Molecular profile of cochlear immunity in the resident cells of the organ of Corti. *Journal of Neuroinflammation*.

[43] Mertins P., NCI CPTAC, Mani D. R. (2016). Proteogenomics connects somatic mutations to signalling in breast cancer. *Nature*.

[44] Li K., Ching D., Luk F. S., Raffai R. L. (2015). Apolipoprotein E enhances microRNA-146a in monocytes and macrophages to suppress nuclear factor-*κ*B-driven inflammation and atherosclerosis. *Circulation Research*.

[45] Shavva V. S., Mogilenko D. A., Nekrasova E. V. (2018). Tumor necrosis factor *α* stimulates endogenous apolipoprotein A-I expression and secretion by human monocytes and macrophages: role of MAP-kinases, NF-*κ*B, and nuclear receptors PPAR*α* and LXRs. *Molecular and Cellular Biochemistry*.

[46] Zhang M., Zhao G.-J., Yin K. (2018). Apolipoprotein A-1 binding protein inhibits inflammatory signaling pathways by binding to apolipoprotein A-1 in THP-1 macrophages. *Circulation Journal*.

[47] Yang X., Chen S., Shao Z. (2018). Apolipoprotein E deficiency exacerbates spinal cord injury in mice: inflammatory response and oxidative stress mediated by NF-*κ*B signaling pathway. *Frontiers in Cellular Neuroscience*.

[48] Liu D., Liang J., Zhao M. (2018). Lysine glycation of apolipoprotein A-I impairs its anti-inflammatory function in type 2 diabetes mellitus. *Journal of Molecular and Cellular Cardiology*.

